# Prescription for astigmatic ametropia revisited

**DOI:** 10.5935/0004-2749.20200072

**Published:** 2020

**Authors:** Sidney Julio de Faria-e-Sousa

**Affiliations:** 1 Faculdade de Medicina de Ribeirão Preto, Universidade de São Paulo, Ribeirão Preto, SP, Brazil

**Keywords:** Astigmatism, Refractive errors, Astigmatismo, Erros de refração

## Abstract

The approach to any refractive condition of the eye with regular astigmatism is more complicated than that for myopia or hyperopia alone. It requires familiarity with the complex images collectively identified as Sturm’s conoid. Fortunately, only three of those play a critical role in the interpretat ion of ametropia with astigmatism. This manuscript discusses a prescription strategy for ametropias associated with regular astigmatism evolved from those three key images.

## Abstract (pt)

A abordagem de qualquer condição refrativa do olho com astigmatismo regular é mais complicada do que a da miopia ou hipermetropia isoladamente. Requer familiarida de com as imagens complexas coletivamente identificadas como o conóide de Sturm. Felizmente, apenas três deles desempenham um papel crítico na interpretação da ametropia com astigma tismo. Este manuscrito mostra como uma estratégia de prescrição para as ametropias associadas ao astigmatismo regular pode evoluir a partir dessas três imagens principais.

## ASTIGMOPIA

**Figure 1:**
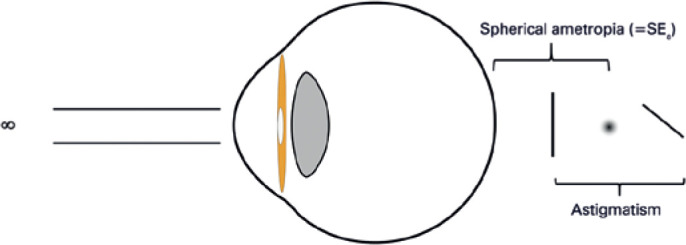
Astigmopia as a combination of a spherical error with astigmatic aberration.

Astigmatic ametropia is a refractive condition of the eye caused by the combination of regular astigmatism with emmetropia or a spherical refractive error, such as myopia or hyperopia (Figure 1). Astigmatism is an ambiguous term that refers either to an aberration or refractive error. To avoid confusion, we coined the term astigmopia [ a (without) + stigma (point) + *ops* (sight) + *íα* ] for the astigmatic ametropia and maintained it unchanged for the aberration^(1)^.

Regular astigmatism is the ensuing aberration following the passage or reflection of light from a toric interface. In a toric interface, the meridional power varies regularly between two principal meridians set 90° apart from each other. A typical example of a toric surface is that of a doughnut^(2)^. Concerning irregular astigmatism, the random variation of the meridional power along the optic interface leads to bizarre optical images that cannot be neutralized with spectacle lenses^(3)^. Consequently, the propositions of the present discussion do not apply to any form of astigmatism other than the regular one.

**Figure 2:**
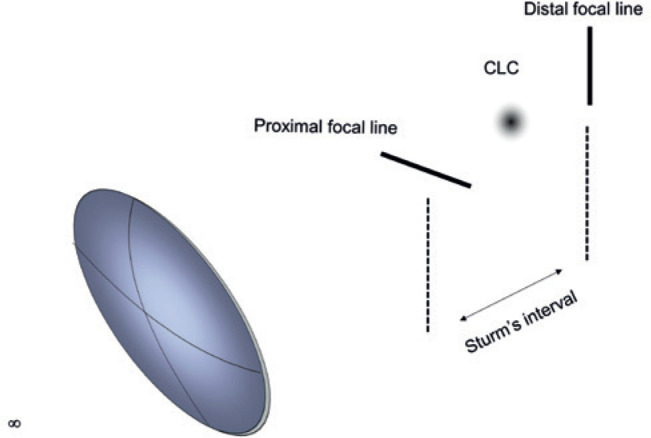
Sturm’s interval and its three main elements.

**Figure 3:**
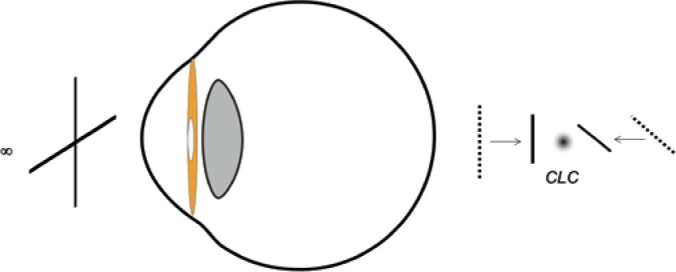
Cross cylinder correction. This approach collapses the Sturm’s interval by approximating both focal lines symmetrically without disturbing the position of the CLC.

For each point-object at infinity, refraction through a toric interface generates two focal lines perpendicular to each other and separated by a variable distance, along the principal axis of the optical system of the eye. The space limited by these lines is termed Sturm’s interval^(2)^. In the diopter center of Sturm’s interval, stands a circular blur termed the circle of least confusion (CLC) (Figure 2). Unlike the focal lines that are in sharp focus in a specific direction, the CLC is an unfocused blur consisting of a cluster of homogeneously scattered points. By lacking directional bias, the CLC is the site of the astigmatic system that best reproduces the shape of the light source^(3)^. This is the reason the spherical equivalent - the spherical lens that puts the CLC on the retina - is so popular among those who deal with astigmatism in eyeglasses, contact lenses, corneal topography, cataract surgery, refractive surgeries, and cross-linking. In theory, placing the CLC on the retina is the best option available to improve vision after deciding to leave astigmatism unchanged^(4)^ (Figure 3).

CLC= circle of least confusion.

### Spherical equivalent

The spherical equivalent of astigmopia (SE_a_) is the spherical power that places the CLC on the retina without altering the amount of astigmatism. It typifies and quantifies the spherical ametropia (myopia or hyperopia) entrenched in astigmopia.

Given any spherocylindrical combination, the SE_a_ is calculated by adding algebraically half of the cylinder power to the power of the associated sphere. For instance, the spherical equivalent of +5.0 ¤ -3.0 cyl 180° is:


$$SE_{a}=\frac{-3.0}{2}+5.0=+3.5D$$

It is crucial to realize that the +5.0 D in the above prescription is the sphere that combined with a cylinder of -3.0 cyl 180° generates the power that neutralizes astigmopia. The real spherical error of the combination is +3.5 D.

It is implicit in the concept of the SE_a_ that the referential point to situate the Sturm’s interval relative to the retina is the CLC, rather than the focal lines that configure the interval. However, the traditional classification of astigmatism is based on the latter assumption, i.e., on the position of the focal lines relative to the retina^(4)^. Hence, when using the spherical equivalent, we are not abiding by the traditional classification and vice-versa. For example, the mixed astigmatism of the traditional classification -with Sturm’s interval straddling the retinaadmits the presence of a CLC in front, on, or behind the retina.

In prescriptions for spherical refractive errors, it is critical to know the dioptric position of the focus relative to the retina. The same reasoning should hold for astigmopia. In practice, there is a consensus that the element that emulates a focus for spherocylindrical combi nations is the CLC, with its dioptric position relative to the retina expressed by the SE_a_.

Given that the traditional classification of astigmopia does not consider the SE_a,_ we proposed a new classification based on the CLC^(5)^. Accordingly, astigmopia is myopic, neutral, or hyperopic when the CLC is in front, on, or behind the retina, respectively. Similarly, a negative, zero, or positive SE_a_ indicates myopia, (spherical) emmetropia, or hyperopia, respectively. The classification of astigmopia based on the CLC is the core element of the prescription strategy described below.

#### Placing the image on the retina

As a rule, we correct myopia entirely to improve vision and under-correct hyperopia to avoid conflict with accommodation. In astigmopia, we use the same approach, by fully neutralizing the myopic, and partially correcting the hyperopic component of ametropia. For instance, after calculating the spherical equivalent of -3.0 ¤ -3.0 cyl 180° and concluding that it is myopic astigmopia (SE_a_ <0), we prescribe full correction to place the image on the retina and maximize vision. We do not anticipate any conflict with accommodation because the eye was not accommodating before. Conversely, after concluding that +3.0 ¤ -3.0 cyl 180° is a hyperopic astigmopia (SE_a_ >0), we consider an under-correction of hyperopia. A full prescription would focus the image on the retina of an eye that already did it by accommodating +1.5 D. By forcing this eye to completely and abruptly relax accommodation, we may cause vision blur due to the usual inability of the ciliary muscle to promptly adapt to this new condition.

We may under-correct the hyperopia as follows: first, we place the image on the retina by prescribing the full astigmopia; then, we discount (subtract) from this provisional prescription the power we want the eye to accommodate. In the last example, if we were going to leave an accommodative effort of + 1.0 D we would subtract this value from +3.0 ¤ -3.0 cyl 180°, leading to the final prescription of +2.0 ¤ -3.0 cyl 180°.

#### Partial correction of astigmatism

Sometimes clinicians decide on a partial correction of astigmatism. It is implicit in this approach a vision degradation proportional to the size of residual astigmatism.

To correct astigmatism is to collapse Sturm´s interval.^(4)^ A plus cylinder brings the distal focal line toward the proximal focal line while a minus cylinder does the reverse. In both cases, the CLC changes its position relative to the retina accompanying the displacement of the focal lines. Consequently, for each amount of residual astigmatism, a new SE_a_ must be calculated before deciding which sphere will place the image on the retina.

A way to avoid the CLC displacement is to neutralize astigmatism with a cross-cylinder, which is the combination of two cylinders of the same power and opposite sign. This combination collapses the Sturm’s interval by approaching both focal lines symmetrically without displacing the CLC^(1)^. In a cross-cylinder, the sphere is always one-half the cylinder power with the inverted sign. For instance, the prescriptions +1.0 ¤ -2.0 cyl 180° and -1.0 ¤ +2.0 cyl 180° are cross-cylinders.

The great advantage of using cross-cylinders is that it allows us to work independently with the spherical and astigmatic components of astigmopia. After determining the cross-cylinder that expresses the amount of astigmatism to be corrected, we add it to the spherical equivalent of astigmopia to find the prescription that places the CLC on the retina. Next, we decide if the image stays in place or should be transferred to anywhere behind the eye, to leave room for some amount of accommodation, as explained earlier.

Let us see an example. What would be the best prescription for a young patient with an astigmopia of +3.0 ¤ -3.0 cyl 180º, in both eyes, if we decide to neutralize only two-thirds of astigmatism and leave +1.0 D of accommodative effort? The solution to this problem involves four steps:

1. Calculate the spherical equivalent of astigmopia, which is SE_a_= +1.5 D
2. Convert two-thirds of astigmatism (-2.0 D) in a crosscylinder, leading to + 1.0 ¤ -2.0 cyl 180º.
3. Add the spherical equivalent to the cross-cylinder, leading to +2.5 ¤ -2,0 cyl 180º. This spherocylindrical combination is the power that places the image on the retina.
4. Subtract the power you want the eye to accommo-date (+1.0 D) from the previous prescription, leading to +1.5 ¤ -2.0 cyl 180º, which is the answer to the problem. This prescription leaves a residual error of +1.50 ¤ -1.0 cyl 180º with SE_a_= +1.0 D.

#### Spherocylindrical equivalent

The prescription that combines the spherical equivalent with the cross-cylinder of the astigmatic correction is termed the spherocylindrical equivalent of astigmopia. It is the power that concomitantly neutralizes the desired amount of astigmatism and places the CLC on the retina.

Representing an astigmopia by S ¤ C cyl α° and the intended correction of astigmatism by C_p_ cyl α°, the spherocylindrical equivalent can be expressed by:


$$\left(S+\frac{C}{2}\right)+\left(-\frac{C_{p}}{2}\text{ व }C_{p}\text{ cyl }\alpha^{\circ }\right)$$

where the first term represents the spherical equivalent and the second, the cross-cylinder of the intended correction of astigmatism. By rearranging relations, we end up with


$$S+\frac{c-C_{p}}{2}\text{ व }C_{p}\text{ cyl }\alpha^{\circ }$$

where S, C, and α° are the sphere, cylinder, and angle that characterize astigmopia, respectively. C_p_ is the intended amount of correction of astigmatism and C-C_p_ is the residual astigmatism.

The last equation is a shorthand for finding the spherocylindrical equivalent of an astigmopia without having to calculate the cross-cylinder of the astigmatic correction. It states that to prescribe the spherocylindrical equivalent, we start by writing the amount of astigmatism to be prescribed (C_p_ cyl α°) and, then, add one-half of the residual cylinder $\frac{c-C_{p}}{2}$ to the associated sphere (S). Returning to the last example of +3.0 ¤ -3.0 cyl 180º, to prescribe two-thirds of astigmatism (-2.0 D) and simultaneously place the CLC on the retina, we add one-half of the residual cylinder $\frac{\left[-3.0-(-2.0)\right]}{2}=-0,5D$ to the associated sphere (+3.0 D) leading to +2.5 ¤ -2.0 cyl 180.

#### Prescription strategy for astigmopia

Based on previous information, we can build a prescription strategy for astigmopia consisting of three steps as follows:

1. Calculate the spherical equivalent. 

This step characterizes the spherical error of the eye with astigmopia where SE_a_ <0 means myopia; SE_a_= 0 indicates the absence of spherical ametropia, and SE_a_ >0 means hyperopia.

2. Place the CLC on the retina with the provisional prescription of the spherocylindrical equivalent.

The calculation of the spherocylindrical equivalent is necessary only when we decide to prescribe part of astigmatism. With full astigmatism prescriptions, the spherocylindrical equivalent coincides with the full refractive error (full correction of astigmopia).

3. Subtract the power you want the eye to accom-modate from the previous prescription.

To make this decision, focus on the nature and the amount of the spherical equivalent. If SE_a_ ≤0, astigmopia is either of the myopic or neutral types. In this case, it is wise to give precedence to visual acuity by keeping the image on the retina with the full prescription the spherocylindrical equivalent already calculated. If SE_a_ >0, astigmopia is of the hyperopic type. In this instance, we may consider a spherical discount to prevent problems with the ciliary tonus. Since the spherical equivalent now indicates the amount of exerted accommodation as a result of the hyperopic state, it should also influence the size of this discount.

This method is applicable either to the negative or positive transcription of astigmatism. After all, these transcriptions are interchangeable.^(2,6)^

Until the efficacy of asymmetrical accommodation in preserving binocularity is proven, it is safer to stick with the traditional principle of making the same discount in both eyes.^(7,8)^
